# Thoracic Endovascular Aortic Repair with Reverse Aortic Arch Debranching Technique for Ascending Aortic Pseudoaneurysm

**DOI:** 10.3400/avd.cr.25-00128

**Published:** 2026-02-06

**Authors:** Hiroki Tada, Keiwa Kin, Tsubasa Mikami, Kazuma Handa, Junya Yokoyama, Yukitoshi Shirakawa

**Affiliations:** 1 Department of Cardiovascular Surgery, Osaka General Medical Center, Osaka, Osaka, Japan; 2 Department of Cardiovascular Surgery, The University of Osaka Graduate School of Medicine, Suita, Osaka, Japan

**Keywords:** TEVAR, ascending aortic pseudoaneurysm, reverse aortic arch debranching technique

## Abstract

Ascending aortic pseudoaneurysm is a rare but life-threatening complication after cardiac surgery, typically requiring redo open repair with substantial risk. We report a 58-year-old male with an incidental 85-mm ascending pseudoaneurysm discovered during evaluation for recurrent hepatocellular carcinoma. Due to prior sternotomy, cirrhosis, and urgent oncologic need, open surgery was deemed prohibitive. He successfully underwent thoracic endovascular repair with reverse extra-anatomical aortic arch debranching technique. He recovered uneventfully and proceeded to cancer treatment without delay. This case highlights the feasibility of hybrid endovascular strategies for ascending aortic pathology in high-risk patients.

## Introduction

Ascending aortic pseudoaneurysm is a rare but potentially life-threatening complication after cardiac surgery. The standard treatment—redo open aortic graft replacement—is associated with high morbidity and mortality.^1)^ Recently, thoracic endovascular aortic repair (TEVAR), including Zone 0 applications, has emerged as a less invasive alternative.^2,3)^ We report a case of a 58-year-old male with ascending aortic pseudoaneurysm managed through a reverse aortic arch debranching technique in conjunction with TEVAR. The patient provided written consent for the disclosure of case-related data and images.

## Case Report

A 58-year-old male was referred to our department following the incidental detection of an ascending aortic aneurysm during evaluation for recurrent hepatocellular carcinoma (HCC). His medical history included ventricular septal defect repair 28 years earlier, chronic hepatitis C-induced cirrhosis (Child-Pugh A), and prior radiofrequency ablation (RFA) for HCC.

**Fig. 1:**
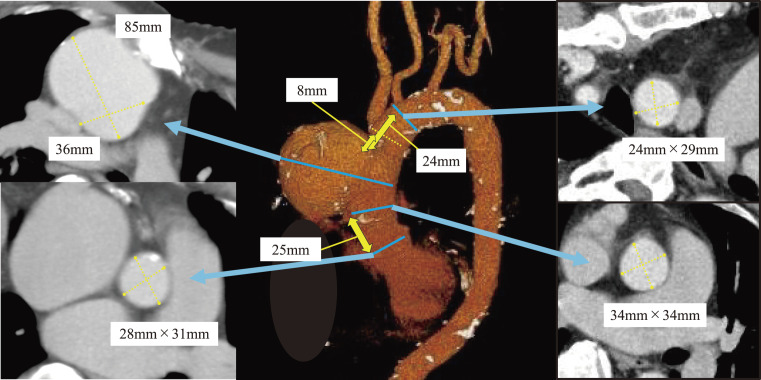
Preoperative CT. A pseudoaneurysm of the ascending aorta measuring 85 × 65 mm was identified. The distance from the STJ to the proximal edge of the pseudoaneurysm was 25 mm, while the distances from the BCA and LCCA to the lesion were 8 and 24 mm, respectively. The aortic diameters at the proximal and distal sealing zones were 34 and 28 mm, respectively, and the aortic wall characteristics were deemed favorable. CT: computed tomography; STJ: sinotubular junction; BCA: brachiocephalic artery; LCCA: left common carotid artery

Contrast-enhanced computed tomography (CT) revealed an 85-mm saccular aneurysm with a neck diameter of 36 mm at the distal ascending aorta, suggestive of a pseudoaneurysm at the previous cannulation site (**Fig. 1**). A persistent left superior vena cava (PLSVC) was also identified. Echocardiography demonstrated preserved left ventricular systolic function (LVEF: 69%) but severe tricuspid regurgitation (TR), and reduced right ventricular function (TAPSE: 13 mm).

Given his prior sternotomy, cirrhosis, venous anatomical anomaly, and right ventricular dysfunction, open repair was deemed high risk (the calculated EuroSCORE II was 9.14%). Prompt TEVAR was chosen to facilitate timely HCC treatment. Preoperative CT showed that debranching of the brachiocephalic artery (BCA) would allow sufficient distal sealing.

Under general anesthesia, small incisions were made in the bilateral subclavian and bilateral cervical regions to encircle the left subclavian artery (LSCA), the right subclavian artery (RSCA), the left common carotid artery (LCCA), and the right common carotid artery (RCCA) with vessel loops. An 8-mm T-shaped Propaten graft (W. L. Gore & Associates, Flagstaff, AZ, USA) was tunneled subcutaneously across the chest, with the RCCA limb routed beneath the clavicle. A prosthetic vascular graft bypass was established from the LSCA to the RSCA and LCCA to the RCCA to ensure long-term patency of the graft. We inserted an S-size Safari guidewire (Boston Scientific, Marlborough, MA, USA) into the left ventricle. A contrast catheter inserted via the left femoral artery enabled angiography of the ascending aorta. A cTAG (W. L. Gore & Associates) stent graft (37 × 37 × 100 mm) was deployed from the distal side (Zone 1) under rapid ventricular pacing (180 bpm), with precise positioning to prevent migration into the aneurysm. Hemodynamics were maintained at 100–120 bpm post-deployment. A second cTAG stent graft (40 × 40 × 100 mm) was then deployed proximally (Zone 0A to Zone 0C) under similar pacing, without ballooning.

Due to inadequate distal sealing, a third cTAG stent graft (37 × 37 × 100 mm) was deployed (Zone 0B to Zone 1), with partial ballooning using a Trilobe balloon under rapid pacing (180 bpm). Final angiography showed no endoleak; however, the third cTAG slightly overlapped the ostium of the LCCA, resulting in delayed LCCA blood flow. Therefore, we prompted deployment of a VBX (W. L. Gore & Associates) stent graft (8 × 39 mm) via LCCA, extending approximately 1 cm into the aorta to secure perfusion.

**Fig. 2:**
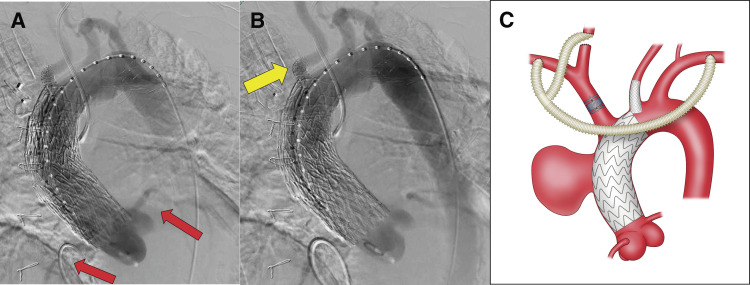
Operative findings. (**A**, **B**) Intraoperative angiography. Coronary perfusion was preserved (red arrows, **A**). Flow through the LCCA was maintained via a VIABAHN stent graft (yellow arrow, **B**). The bypass graft patency was confirmed, and no Endoleak was observed (**B**). (**C**) Schematic illustration of the surgical technique. LCCA: left common carotid artery

To prevent type II endoleak, a 20-mm Amplatzer Vascular Plug II (AGA Medical, Golden Valley, MN, USA) was placed at the BCA origin. Completion angiography confirmed aneurysm exclusion and graft patency (**Fig. 2A** and **2B**). The finished schematic is shown below (**Fig. 2C**).

**Fig. 3:**
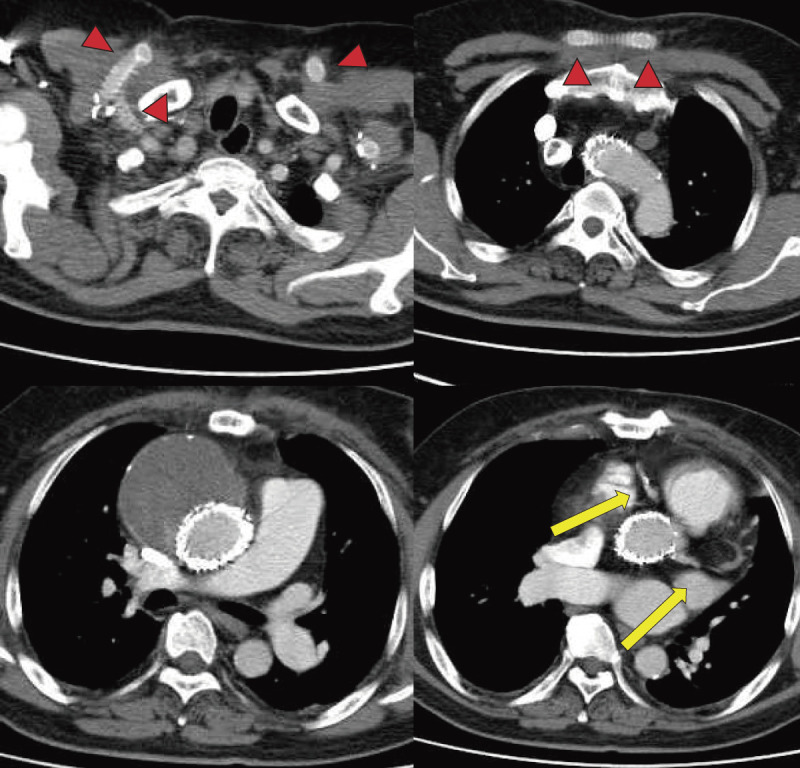
Postoperative CT. The graft remained patent (red arrowheads), and no endoleak was identified. Both right and left coronary arteries were well opacified, confirming preserved coronary flow (yellow arrows). CT: computed tomography

The patient was extubated in the operating room. Postoperative CT showed no endoleak or bypass graft dysfunction (**Fig. 3**). The patient had no neurological deficits and underwent RFA for recurrent HCC without delay. The patient died 1 year later due to liver failure, with no aortic events observed during the follow-up period.

## Discussion

Although pseudoaneurysm of the ascending aorta following open cardiac surgery is rare, it remains a potentially fatal complication, typically occurring at sites such as the aortic cannulation site, aortotomy, or prosthetic graft anastomosis.^4)^ Redo sternotomy carries high risk, with mortality rates reported up to 17%.^1)^ In contrast, TEVAR for ascending aortic diseases has shown favorable outcomes, with technical success rates above 95% and 30-day mortality around 3%.^5)^

Currently, several anatomical and technical challenges still limit the broader adoption of ascending TEVAR. Anatomically, the ascending aorta is flanked by critical structures—the aortic valve and coronary ostia proximally, and the supra-aortic branches distally—making it difficult to secure an adequate sealing zone. Consequently, the current optimal indications for ascending TEVAR are limited to localized lesions such as pseudoaneurysms or dissections, particularly those confined between Zone 0B and Zone 0C. The average length of the ascending aorta is 7–8 cm, which is often insufficient for currently available devices that lack conformability to curvature. Technically, deploying a stent graft in the ascending aorta is difficult due to short landing zones and direct exposure to pulsatile cardiac output, making precise deployment challenging. Although the transsubclavian and transapical approaches have been reported to be advantageous for accurate deployment due to shorter working distances, the transsubclavian approach has limitations in vessel diameter and tortuosity, while the transapical approach requires thoracotomy.^6,7)^ In our case, regarding the transsubclavian approach, the presence of an anastomosis at the access site rendered it unsuitable, while the transapical approach was precluded by a history of sternotomy. Therefore, we were obliged to select the TF approach, and we employed rapid ventricular pacing to minimize stent graft migration. Furthermore, there was a concern that the device’s nose cone might interfere with the aortic valve. We selected the cTAG stent graft with a short nose cone and consider the device useful for avoiding aortic valve insufficiency.

In the present case, a reverse debranching of the BCA was required to obtain a sufficient distal sealing zone, necessitating procedural innovation. Several extra-anatomic bypass techniques have been reported in the literature for ascending aortic TEVAR. For example, Shimizu et al.^8)^ reported the LSCA to the LCCA, the RCCA, and the RSCA with descending aorta–LSCA bypass, which requires left lateral thoracotomy and single-lung ventilation, making it highly invasive. Although Gomibuchi et al.^9)^ reported the right common femoral artery–BCA bypass, which is technically simpler, it involves a long retrograde course against gravity, raising concerns about long-term patency and an increased risk of cerebral embolism in patients with diseased aortas. Canaud et al.^6)^ reported a case of ascending TEVAR using “reverse” debranching technique bypasses from the LSCA to the RCCA and RSCA, similar to our approach. This technique avoids thoracotomy and is effective; however, the retropharyngeal graft route carries the risk of long-term pharyngolaryngeal dysfunction^10)^ and potential extension to mediastinitis in the event of graft infection. Our subcutaneous thoracic extra-anatomic bypass was less invasive, technically straightforward, and demonstrated favorable expectations for graft patency. Nonetheless, because the LSCA served as the inflow for BCA debranching, evidence regarding long-term patency is limited, warranting careful surveillance, although Shimizu et al. reported that cerebral blood flow would be sufficient.

In this patient, urgent intervention was required due to the local recurrence of HCC. Minimizing systemic deterioration was essential to allow rapid resumption of cancer therapy. Postoperatively, the patient maintained functional status and was able to proceed to HCC treatment promptly. Although hybrid repair is not yet a complete substitute for surgical repair of ascending aortic pathology due to anatomical complexity, lack of dedicated devices, deployment challenges, and insufficient long-term outcome data, our procedure demonstrated its clinical value, particularly in high-risk patients who require rapid recovery and early treatment for concomitant disease.

## Conclusion

We successfully performed TEVAR combined with a reverse extra-anatomical aortic arch debranching procedure for an ascending aortic pseudoaneurysm, achieving favorable outcomes. While further studies are warranted, this case highlights that hybrid repair with reverse arch debranching can expand the therapeutic options for high-risk patients with ascending aortic pathology.
